# Phenotypic and genotypic characterization of *Candida* species isolated from candideamia in Iran

**DOI:** 10.18502/cmm.4.2.64

**Published:** 2018-06

**Authors:** Seyedeh Zahra Sadrossadati, Mohammad Ghahri, Abbas Ali Imani Fooladi, Shirin Sayyahfar, Sedigheh Beyraghi, Zohre Baseri

**Affiliations:** 1 Department of Biology, Ashkezar Branch, Islamic Azad University, Iran; 2 Department of Biology, School of Applied Sciences, Imam Hossein University, Tehran, Iran; 3 Applied Microbiology Research Center,Systems Biology and Poisonings Institute, Baqiyatallah University Medical of Sciences, Tehran, Iran; 4 Department of Pediatrics, Division of Pediatric Infectious Diseases, Ali Asghar Children Hospital, Research Center of Pediatric Infectious Diseases, Institute of Immunology and Infectious Diseases, Iran University of Medical Sciences, Tehran, Iran; 5 Department of Medical Technology, School of Medicine, Tehran University of Medical Sciences, Tehran, Iran; 6 Department of Pathology and Laboratory Medicine, Shariati Hospital, Tehran University of Medical Sciences, Tehran, Iran

**Keywords:** Candidemia, Blood culture, Epidemiology, PCR-RFLP

## Abstract

**Background and Purpose:**

Candidemia is one of the most important fungal infections caused by *Candida* species. Infections and mortality caused by *Candida *species have been on a growing trend during the past two decades. The resistance of yeasts to antifungal drugs and their epidemiological issues have highlighted the importance of accurately distinguishing the yeasts at the species level. The technique applied for yeast identification should be fast enough to facilitate the imminent initiation of the appropriate therapy. Candidemia has not been studied comprehensively in Iran yet. Regarding this, the current study aimed to assess the epidemiology of candidemia at Tehran hospitals and compare the results with the previous ﬁndings.

**Materials and Methods:**

This study was conducted on 204 positive blood cultures obtained from 125 patients hospitalized in several hospitals located in Tehran, Iran, within a period of 13 months. The yeast isolation and species identification were accomplished using several phenotypic methods (i.e., production of germ tube in human serum, culture on CHROMagar *Candida*, and Corn meal agar containing Tween 80) and molecular methods, such as polymerase chain reaction-restriction fragment length polymorphism (PCR-RFLP). In addition, unknown cases were subjected to PCR sequencing. These methods were then compared in terms of accuracy, sensitivity, and speed of identiﬁcation.

**Results:**

According to the results, *C. albicans* (62.4%) was the most common isolate, followed by *C. parapsilosis* (n=36, 17.5%), *C. glabrata* (n=18, 8.8%), *C. tropicalis* (n=13, 6.3%), *Trichosporon asahii* (n=3, 1.5%), *C. kefyr* (n=2, 1.0%), *C. lusitaniae* (n=2, 1.0%), *C. intermedia* (n=1, 0.5%), *C. guilliermondii* (n=1, 0.5%), and *C. krusei* (n=1, 0.5%), respectively.

**Conclusion:**

As the findings indicated, the most common species causing candidemia were *C. albicans*, *C. parapsilosis*, and *C. glabrata*, respectively. Children less than one year old and people with cancer were at higher risk for candidemia, compared to other groups. Moreover, phenotypic and molecular methods resulted in the identification of 65.2% and 96.6% of the isolates, respectively. Consequently, PCR-RFLP could be concluded as a more favorable technique for species identification.

## Introduction

Candidemia is one of the most important fungal infections caused by *Candida* species [1, 2]. Deaths due to *Candida* infections have been on a growing trend during the past two decades [1]. According to the literature, the patients admitted to the Intensive Care Units have a higher rate of mortality caused by candidemia, compared to others (from 60% to 80%) [1-6]. Given the resistance of the yeast to antifungal drugs and their epidemiological issues, the species-level identify-cation of the yeast should be performed with a high accuracy [1, 2, 7-10]. In addition, the detection methods should be fast enough in order to facilitate the eminent initiation of the appropriate therapy. Therefore, there is an increasing demand to improve the detection ability and speed of the methods employed for the isolation and detection of the yeasts [1, 4, 5, 7, 10, 15].

In our previous study, which was conducted in one of the greatest hospitals of Tehran, Iran, during Aug. 2008 to Nov. 2009, *C. parapsilosis* was identified as the most common isolate obtained from candidemia patients [7]. Therefore, we decided to expand our study by investigating different large hospitals (i.e., perform a multicenter study) to ﬁnd more accurate results, especially from the epidemiologic aspect. 

With this background in mind, the present study was conducted to determine the common *Candida* species in Tehran hospitals. To this end, we managed to isolate yeasts from the blood cultures of the patients suffering from candidemia in several hospitals of Tehran. Several traditional techniques and some new molecular methods (e.g., polymerase chain reaction-restriction fragment length polymer-phism [PCR-RFLP]) were performed to identify *Candida* species. Moreover, as a secondary goal of this research, we compared several *Candida* identiﬁcation methods based on different metrics. In particular, we compared the experimental results in terms of the accuracy, sensitivity, and speed of each method, and provided a detailed discussion regarding the more effective identiﬁcation method by considering all aspects.

## Materials and Methods

A total of 40,412 blood samples obtained from patients admitted to nine hospitals of Tehran (i.e., Imam Khomeini, Children's Medical Center, Baqiyatallah, Ali Asghar, Shariati, Tehran Heart Center, Shohadaye Tajrish, Rasoul Akram, and Firoozgar) were cultured within February 2014 to March 2015. The study was approved by the Medical Research Ethics Committee of Yazd University of Medical Sciences, Yazd, Iran (IR.IAU.YAZD.REC.1397,23). In order to isolate yeast colonies from blood cultures, the blood cultures were subcultured on Sabouraud dextrose agar (Merck, Germany) and incubated at 25-30°C for 48 h. Subsequently, the colonies were transferred into tubes containing 35% glycerol in distilled water and kept at 20°C for further study [7]. In this study, six standard strains, including *C. albicans* (ATCC 10231), *C. parapsilosis* (ATCC 90018), *C. glabrata* (ATCC 90030), *C. krusei* (ATCC 6258), *C. tropicalis* (ATCC 0750), and *C. lusitaniae* (JCM 1619), were employed as quality controls.

The yeasts were ﬁrst identiﬁed according to morphological characteristics and physiological properties using several phenotypic techniques, such as the production of germ tube in human serum [1, 7, 11], culture on CHROMagar *Candida *(BioMerieux, France) [1, 7, 11, 12], and Corn meal agar (Difco, USA), containing Tween 80 [7, 11, 12]. After the initial morphological identiﬁcation, the extraction of yeast DNA was carried out using the direct PCR and boiling method [1, 16, 17]. We performed PCR-RFLP based on a standard method described by Mirhendi et al. [13, 14, 18]. In the mentioned study, universal primers, namely ITS1 (5-TCCGTAGGTGAACCTGCGG-3) (CinnaGen, Iran) and ITS4 (5-TCCTCCGCTTATTGATATGC-3) (CinnaGen, Iran), were used to facilitate the ampliﬁcation of the ITS1-5.8S rRNA ITS2 regions. 

The PCR ampliﬁcation was performed in a ﬁnal volume of 50 µL. Each reaction contained 1 µL template DNA, 0.5 µM of the primers, 1.5 mM MgCl_2_ (Sinaclon, Iran), 400 µM of deoxynucleoside triphosphate (dNTP) (Sinaclon, Iran), 5 µL of 10x PCR buffer (Sinaclon, Iran), and 1.25 U of Taq DNA polymerase (Fermentas, Lithuania). The PCR process included an initial denaturation step at 94°C for 5 min, followed by 35 cycles of denaturation at 94°C for 30 sec, annealing at 56°C for 45 sec with an extension of 72°C for 1 min, and a ﬁnal extension step at 72°C for 7 min. 

The presence of speciﬁc 400-900 base-pair PCR products was examined through staining with ethidium bromide (Sinaclon, Iran) after electro-phoresis on 1.5% agarose gel (Promega, USA). The ampliﬁed PCR products were digested with the restriction enzyme *MspI *(Thermo Fisher Scientiﬁc, USA) in order to distinguish the common yeast species, including *C. albicans*, *C. tropicalis*, *C. glabrata*, *C. parapsilosis*, and *C. krusei* [1, 18]. The remaining unknown yeasts (n= 6, 3.4%) were sent for DNA sequencing. 

## Results

**Figure 1:**
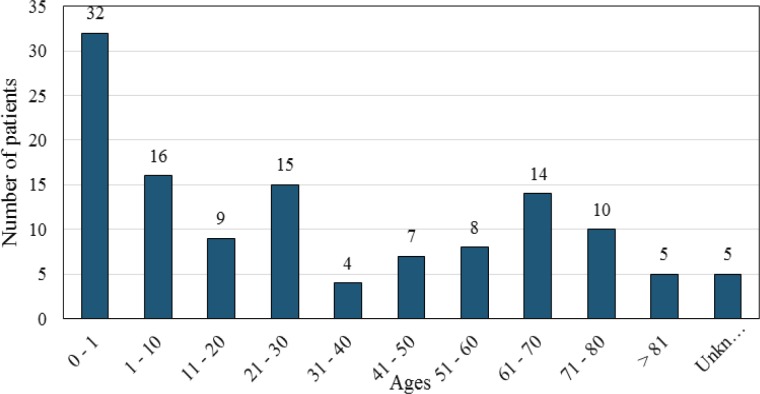
Number of patients with candidemia based on different age groups

Out of the 40,412 tested samples, 204 cases were positive for yeasts. The positive blood samples belonged to 125 patients, including 72 females (57.6%) and 53 males (42.4%) with the mean ages of 36.1 and 20.7 years, respectively. The age range of the patients was 0-90 years. Figure 1 depicts the effect of patients' age on the frequency of *Candida* species occurrence in blood cultures. Our comprehensive study of the medical records of patients showed that all patients with candidemia had an underlying disease (see Table 1). 

**Figure 2:**
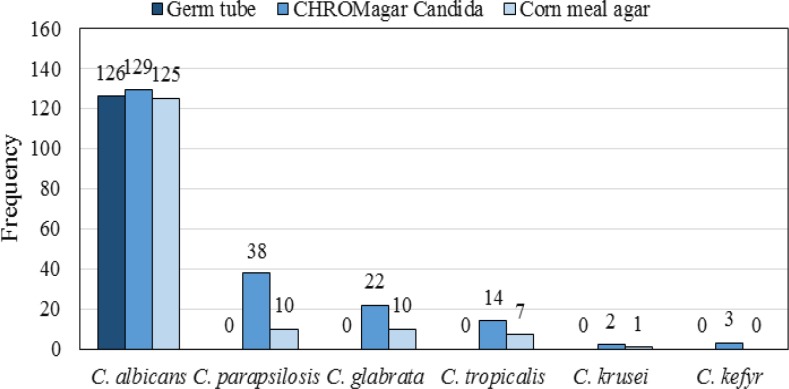
Comparison of phenotypic tests with respect to the number of isolated species

**Figure 3:**
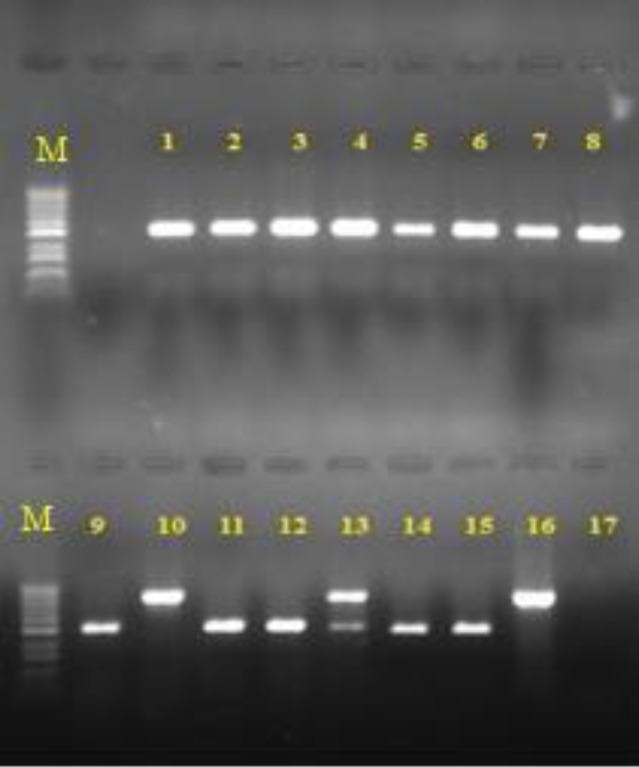
*Electrophoresis of polymerase chain reaction products; lanes 1-9, 11, 12) *C. albicans* (535 bp), lanes 14 and 15) *C. parapsilpsis* (520 bp), lanes 10 and 16) *C. glabrata* (871 bp), lane 13) a combination of *C. albicans* and *C. glabrata*, lane 17) negative control, and lane M) 100 bp DNA size marker*

**Figure 4:**
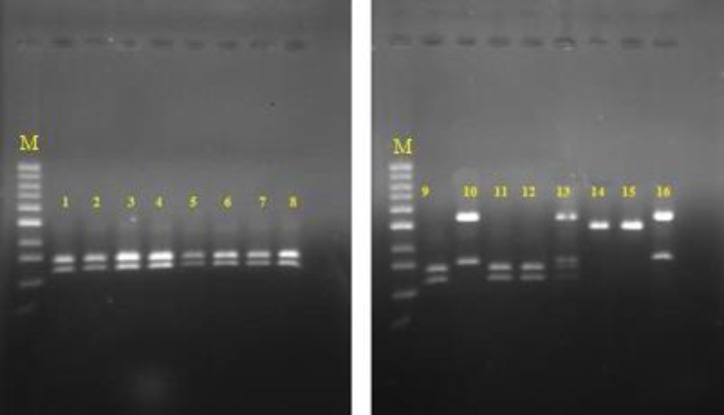
Polymerase chain reaction-restriction fragment length polymorphism proﬁle of samples; lanes 1-9, 11, 12) *C. albicans* (297, 238 bp), lanes 14, 15) *C. parapsilpsis* (520 bp), lanes 10, 16) *C. glabrata* (557, 314 bp), lane 13) a combination of *C. albicans* and *C. glabrata*, and lane M) 100 bp DNA size marker

**Figure 5:**
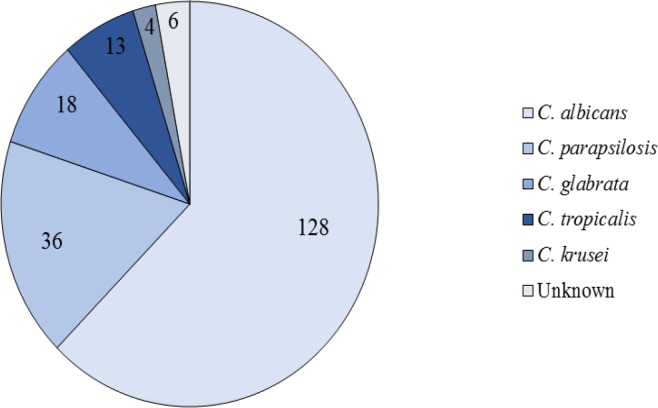
Frequency of the species isolated from the blood cultures of patients with candidemia using polymerase chain reaction-restriction fragment length polymorphism

**Figure 6:**
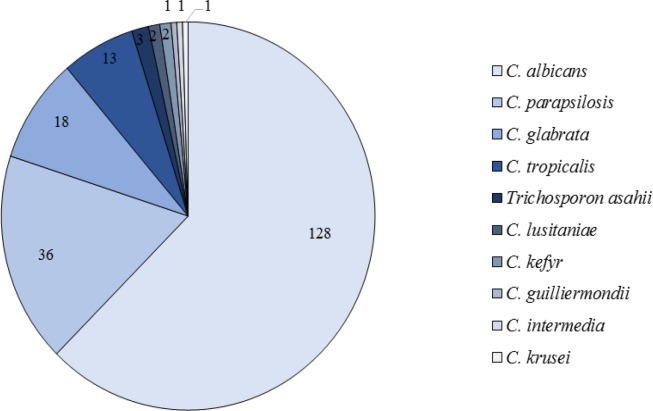
Overall frequency of the species isolated from the blood cultures of patients with candidemia

Other predisposing factors for candidemia in our experiments were the use of antibiotics, antifungal drugs, and corticosteroid, as well as surgery. Table 2 presents the different predisposing factors and their frequency. According to the available information, 45 patients (36%) passed away in these hospitals. Figure 2 illustrates the results of different phenotypic methods used for the identification of *Candida* species in positive blood cultures. The electrophoresis of PCR products is displayed in Figure 3. Figure 4 demonstrates the RLFP-PCR profiles of the samples using MspI restriction enzyme. Figure 5 reports the frequency of the species obtained by using PCR-RFLP. As can be seen in Figure 5, six cases remained still unknown after the implementation of PCR-RFLP. As a result, these unknown cases, along with three uncertain cases as control specimens, were subjected to DNA sequencing. Figure 6 illustrates the contribution of each* Candida* species in the positive blood cultures.

**Table 1:** Frequency of different underlying diseases in patients with candidemia

**Underlying Diseases**	**Frequency (%)**
Infections caused by iatrogenic factors	1.8
Impaired immune system (e.g., neutropenia, lupus, and Parkinson)	1.8
Liver diseases	2.7
Cardiac diseases	3.5
Bacterial and viral infections	4.4
Digestive disorders	5.3
Diabetes	5.8
Renal failure	7.1
Pulmonary disorders	8.0
Metabolic and genetic disorders	9.7
Brain and neurologic	11.1
Sepsis	11.9
Cancer: Hematologic malignancy	2.7
Cancer: Other tumors	9.7
Other	14.6

**Table 2:** Predisposing factors for candidemia

**Candidemia Predisposing Factors**	**Frequency (%)**
Prematurity	0.9
Surgery	16.0
Organ transplants	2.1
Antibiotics	33.1
Antifungal drugs	18.7
Corticosteroid	12.7
Receiving immunosuppressive therapy	1.5
Chemotherapy	3.9
Iatrogenic illness	11.1

## Discussion

Several methods have been proposed to detect and distinguish *Candida* species in a positive blood culture. It is important to employ an accurate and fast technique for the identification of *Candida* species in order to select a proper therapy imminently [1, 2, 10]. Therefore, in this research, we used three well-known phenotypic methods and two genotypic methods, namely PCR-RFLP and DNA sequencing, to detect *Candida* species in 204 positive blood cultures. This section includes some discussions about our experiments and comparisons of the applied techniques. In the current study, *C. albicans* was recognized as the dominant causative agent of candidemia; in this regard, this species was observed in 62.4% of the tested positive blood cultures. 

Moreover, *C. parapsilosis* (17.5%) and *C. glabrata* (8.8%) were the second and third common species detected in our evaluated positive blood cultures, respectively. In a study performed by Ghahri et al. (2012) [7], *C. parapsilosis* was reported as the most common species identified in candidemia patients admitted to Baqiyatallah Hospital, Tehran, Iran. Therefore, the current research was initiated to holistically study the different etiologic agents of candidemia in patients hospitalized in several important hospitals of Tehran.

This is in line with the results reported from different regions of the world, such as those obtained by Nieto-Rodriguez et al. [19] in the USA, Yildiz et al. [6] in Turkey, Wille et al. [2] in Brazil, and Razzaghi et al. [20] in Kashan, Iran. In the present study, *C. parapsilosis* and *C. glabrata *had the second and third ranks of occurrence, respectively. On the other hand, in the study of Razzaghi et al. [20], *C. glabrata* and *C. parapsilosis* had the second and third ranks, respectively. This difference is mainly due to the investigation of different hospitals and the number of positive blood cultures in these two studies. Therefore, the results achieved in the previous studies conducted in Tehran are limited to that period and speciﬁc location.

In the current study, out of the 40,412 blood cultures sent to the laboratory, 204 cases (0.51%) were positive. This ratio was also obtained in our previous study performed on 5,141 blood cultures. Regarding this, it could be concluded that the rate of candidemia occurrence in patients admitted to Tehran hospitals has remained almost unchanged from 2011 to 2017. In the current study, PCR-RFLP method failed to identify six isolates (3.4%); therefore, they were subjected to DNA sequencing.

As indicated in Figure 2, CHROMagar *Candida* showed a better function in species identification than other phenotypic methods used in this study. This method facilitated the detection of *Candida* species in 84.8% of the positive blood cultures. Accordingly, it resulted in the detection of *C. albicans* (63.2%), *C. parapsilosis* (18.6%), *C. glabrata* (10.8%), *C. tropicalis* (6.9%), and *C. krusei* (0.9%) in the positive blood cultures. Moreover, in the current study, there were some positive blood cultures having more than one *Candida* species. CHROMagar *Candida* is the only phenotypic method that can detect the mixed cultures of *Candida* species in the positive blood cultures. 

As an example, we found combinations of *C. albicans* and *C. glabrata*, as well as *C. glabrata* and *C. parapsilosis* three and four times, respectively. The aforementioned discussion showed that CHROMagar *Candida* outperformed other phenotypic methods in terms of the ability of *Candida* species detection. In the present study, phenotypic methods resulted in the detection of *Candida* species in 65.2% of the isolates. However, only 3.4% of the isolates remained unknown when using the PCR-RFLP. The results of PCR-RFLP were consistent with those of the phenotypic methods in 97.5% of the cases. This clearly conﬁrmed the efficiency of the phenotypic methods. In terms of the speed of detection, PCR-RFLP outperformed other phenotypic methods as it could be performed in about 5 h, while other phenotypic methods needed about 48-72 h.

Considering the epidemiologic aspects, most of the patients with candidemia were female (57.6%) with a mean age of 36.1 years. The analysis of the age of the patients with candidemia revealed that children had the highest risk factor for this disease in comparison with other age groups. The* Candida* species detected in children were mostly *C. albicans* (n=49) and *C. parapsilosis* (n=19), respectively. Regarding the adults (i.e., less than 60 years old), *C. albicans*, *C. parapsilosis*, and *C. tropicalis* were identified in 47, 9, and 9 cases, respectively. Furthermore, considering the elderly group (i.e., more than 60 years old), composing 23.2% of the patients with candidemia, similar to other age categories, *C. albicans* was more common than other *Candida* species. The second common *Candida* species in the elderly group was *C. glabrata,* which rarely happened in other age groups. 

In the current study, the most common predisposing factors for candidemia were antibiotic therapy, antifungal drugs, and surgery (Table 1). Moreover, cancer and sepsis were among the high-risk factors for candidemia. This study was performed for the partial fulfillment of the requirements for the degree of Master of Science; consequently, the study had limited time period. Moreover, the current study received no funding support to cover the expenses of performing phenotypic and genotypic methods.

## Conclusion

As the findings of the present study indicated, *Candida albicans* was the most common *Candida* species of candidemia in patients admitted to the hospitals of Tehran, followed by *C. parapsilosis* and *C. glabrata*, respectively. In addition, PCR-RFLP outperformed other phenotypic methods in terms of accuracy and detection speed.
